# Investigating the effects of PTEN mutations on cGAS-STING pathway in glioblastoma tumours

**DOI:** 10.1007/s11060-023-04556-4

**Published:** 2024-01-12

**Authors:** Eda Dogan, Zafer Yildirim, Taner Akalin, Erkin Ozgiray, Nevhis Akinturk, Cagdas Aktan, Asli Ece Solmaz, Huseyin Biceroglu, Kadri Emre Caliskan, Yesim Ertan, Taskin Yurtseven, Buket Kosova, Vildan Bozok

**Affiliations:** 1 Department of Medical Biology, Ege University Faculty of Medicine, Izmir, Türkiye; 2 Department of Pathology, Ege University Faculty of Medicine, Izmir, Türkiye; 3 Department of Neurosurgery, Ege University Faculty of Medicine, Izmir, Türkiye; 4 Department of Medical Biology, Beykent University School of Medicine, İstanbul, Türkiye; 5 Department of Medical Genetics, Ege University Faculty of Medicine, Izmir, Türkiye

**Keywords:** Glioblastoma, PTEN, cGAS-STING pathway, Interferon response, Immunogenicity

## Abstract

**Background:**

*PTEN* is a tumour suppressor gene and well-known for being frequently mutated in several cancer types. Loss of immunogenicity can also be attributed to PTEN loss, because of its role in establishing the tumour microenvironment. Therefore, this study aimed to represent the link between PTEN and cGAS-STING activity, a key mediator of inflammation, in tumour samples of glioblastoma patients.

**Methods:**

Tumour samples of 36 glioblastoma patients were collected. After DNA isolation, all coding regions of *PTEN* were sequenced and analysed. PTEN expression status was also evaluated by qRT-PCR, western blot, and immunohistochemical methods. Interferon-stimulated gene expressions, cGAMP activity, CD8 infiltration, and Granzyme B expression levels were determined especially for the evaluation of cGAS-STING activity and immunogenicity.

**Results:**

Mutant *PTEN* patients had significantly lower PTEN expression, both at mRNA and protein levels. Decreased *STING, IRF3, NF-KB1*, and *RELA* mRNA expressions were also found in patients with mutant *PTEN*. Immunohistochemistry staining of PTEN displayed expressional loss in 38.1% of the patients. Besides, patients with PTEN loss had considerably lower amounts of *IFNB* and *IFIT2* mRNA expressions. Furthermore, CD8 infiltration, cGAMP, and Granzyme B levels were reduced in the PTEN loss group.

**Conclusion:**

This study reveals the immunosuppressive effects of PTEN loss in glioblastoma tumours *via* the cGAS-STING pathway. Therefore, determining the PTEN status in tumours is of great importance, like in situations when considering the treatment of glioblastoma patients with immunotherapeutic agents.

**Supplementary Information:**

The online version contains supplementary material available at 10.1007/s11060-023-04556-4.

## Introduction

Glioblastoma is the most common and aggressive brain tumour that frequently displays the loss of phosphatase and tensin homologue (PTEN) activity, both in primary and recurrent tumour types [1]. PTEN has phosphatase activities on target protein as well as on target lipids, thus regulating a wide range of cellular functions from cell death to motion [2]. Loss of PTEN activity can be caused by gene mutations, epigenetic regulation, or post-transcriptional and -translational regulations. PTEN loss was reported in 55.9% of gastric cancer cases with *PTEN* mutations [3]. *PTEN* mutations have a negative impact on survival and are reported around 40% and 33% in all glioblastoma and IDH wild type glioblastoma cases, respectively [4, 5]. Mutations of genes that regulate oncogenic signalling pathways are associated with immunosuppressive tumour microenvironment [6–8]. GBM studies have shown that programmed death ligand-1 (PD-L1) expression increases after PTEN loss [9] and *PTEN* is significantly mutated in anti-PD-1 immunotherapy non-responsive tumours [10].

cGAS and STING are cytoplasmic DNA sensors and key regulatory proteins triggering innate immune response [11]. After activation and transmission of their signal to the nucleus *via* the TANK-binding kinase 1 (TBK1) and Interferon regulatory factor 3 (IRF3) proteins, transcription of type I interferon (IFN) and interferon-stimulated genes (ISGs) are activated, which in turn regulate many innate and adaptive immune system cells. The increase of cytosolic chromatin fragments and micronuclei along with replicative stress during malignant transformation increases the possibility of DNA leakage in cancer cells and thus activates the cGAS-STING pathway [12].

It has been shown that PTEN has protein phosphatase activity on IRF3, which is the transcription factor of type I IFN. Therefore, PTEN provides a direct link between tumour suppression and antiviral innate immunity [13]. If PTEN displays antitumor activity, in part through the stimulation of type I IFN response, its loss might disrupt the cGAS-STING pathway and contribute to the immunosuppressive microenvironment of GBM tumours. Based on this hypothesis, this study aimed to explore the effects of *PTEN* mutations and expressional status on the cGAS-STING activity in glioblastoma tumours.

## Methods

### Patients and tumour samples

Our study protocol was approved by the Institutional Clinical Research Ethics Committee (2018, 18-12.1T/17). Written informed consent was obtained prior to enrolment. Patients diagnosed with primary or recurrent GBM were included into this study. GBM samples were collected during surgical intervention and processed for immunohistochemistry and molecular analysis. All tissue samples were classified based on the WHO classification [14] and IDH-positive samples were excluded from the study. A tissue aliquot was used for DNA, RNA, and protein purification; whereas, another aliquot was fixed in a 4% paraformaldehyde solution. Since the Neurosurgery Department of Ege University Faculty of Medicine constitutes the reference clinic of West Turkey, patients were preferentially transferred to other health care centres for further adjuvant treatment and follow-ups, after early follow-up CT scans were taken.

### Next generation sequencing

DNA was isolated from fresh GBM tissue samples with the QIAamp DNA Isolation Kit (Qiagen). All coding sequences of *PTEN* were amplified with 10x specific primer sets. The Nextera DNA Library Preparation Kit (Illumina) was used for library preparation from pooled PCR amplicons and sequenced with the NextSeq 550 System (Illumina). Quality control and evaluation of gene sequencing results were performed by use of appropriate programs as previously described [15].

### Quantitative RT-PCR

Total RNA was isolated from fresh GBM tissue samples with the RNeasy Kit (Qiagen) and 1 µg was transcribed to cDNA by using the iScript Reverse Transcription Supermix Kit (Bio-Rad). qRT-PCR was performed for the cGAS-STING pathway members and interferon-regulated genes using the primer pairs below (5’→3’) and iTaq Universal SYBR Green Supermix (Bio-Rad) on the LightCycler 480 Instrument (Roche). The relative target gene mRNA expression level was normalized to the *GAPDH* housekeeping gene by using the ΔΔCt method.

GAPDH (F: CATTGCCCTCAACGACCACTTT; R: GGTGGTCCAGGGGTCTTACTCC)

GZMB (F: CTTCCTGATACGAGACGACTTC; R: CGGCTCCTGTTCTTTGATATTG)

IFIT2 (F: GCGTGAAGAAGGTGAAGAGG; R: GCAGGTAGGCATTGTTTGGT)

IFI44 (F: GATGTGAGCCTGTGAGGTCC; R: CTTTACAGGGTCCAGCTCCC).

IFNB1 (F: CAGCATCTGCTGGTTGAAGA; R: CATTACCTGAAGGCCAAGGA)

IL6 (F: AGACAGCCACTCACCTCTTCAG; R: TTCTGCCAGTGCCTCTTTGCTG)

IRF3 (F: AGAGGCTCGTGATGTGGTCAAG; R: AGGTCCACAGTATTCTCCAGG).

ISG15 (F: CAGCCATGGGCTGGGAC; R: GCCGATCTTCTGGGTGATCT)

NF-KB1 (F: GAAGCACGAATGACAAGAGGC; R: GCTTGGCGGATTAGCTCTTTT)

PTEN (F: CCACAAACAGAACAAGATGCT; R: GCTCTATACTGCAAATGCTATCG)

RELA (F: TCACCCCCACGAGCTTGTA; R: TTGTTGTTGGTCTGGATGCG).

STING (F: GCAGTGTGTGAAAAAGGGAAT; R: AGGTCCACAGTATTCTCCAGG)

### Western blot

Fifty mg of fresh GBM tissue sample was lysed in RIPA buffer, before 14 µg of the protein lysate was loaded onto a 12% SDS-PAGE gel for separation. PTEN (ProteinTech, 1:2000), STING (D2P2F; Cell Signaling, 1:1000), IRF3 (D6I4C; Cell Signaling, 1:1000), NF-KB (L8F6; Cell Signaling, 1:1000), Phospho-NF-KB (Ser536; 93H1; Cell Signaling, 1:1000), Beta-Actin (Cell Signaling, 1:1000) primary antibodies; and, Anti-Rabbit IgG (Cell Signaling, 1:1000) and Anti-Mouse IgG (Cell Signaling, 1:1000) secondary antibodies were used. The Clarity Western ECL Substrate (Bio-Rad) and C-DiGit Blot Scanner (Licor) were used for the detection of ECL signals.

### cGAMP activation

The 2’3’-cGAMP ELISA Kit was used according to the manufacturer’s instructions (Cayman Chemical). Protein lysates were prepared using RIPA buffer (Thermo Scientific) and Protease inhibitors (Thermo Scientific). In the experiments, 30 µg of protein lysates were used and all samples were run in duplicates.

### Immunohistochemistry

Five µm sections were prepared from formalin-fixed and paraffin-embedded GBM tissue samples, mounted on poly-L-lysine-coated slides, and processed according to a standard immunohistochemical (IHC) procedure as previously described [16]. Slides were incubated with PTEN (ProteinTech, 1:200) or CD8 (Clone 1A5, Novocastra, 1:30) primary antibodies for 32 min at RT. All slides were reviewed by two pathologists (TA and YE). Immunohistochemical expression patterns of PTEN were interpreted in accordance with the literature [17]. CD8 density was evaluated in 3 grades according to the distribution density within the tumour. The presence of CD8 + lymphocyte clusters, i.e. ≥ 20 CD8 + lymphocytes in one HPF within the tumour was evaluated as +++. The presence of ≥ 20 CD8 + lymphocytes per mm^2^ (⁓5 HPF) in the densest area of the tumour was evaluated as ++. If the presence of CD8 + lymphocytes per mm^2^ (⁓5 HPF) in the densest area of the tumour was < 20, it was evaluated as +.

### Bioinformatic analysis

The Cancer Genome Atlas (TCGA) database was used to evaluate *PTEN* gene expression in a total of 162 samples, all selected from Glioblastoma Multiforme and containing *PTEN* expression data (TCGA, Firehose Legacy) [18, 19]. Samples were split into two groups based on median *PTEN* expression (high vs. low). The p-value derived from Student’s t-test and q-value from Benjamini-Hochberg procedure. Tumor IMmune Estimation Resource (TIMER), on the other side, is a comprehensive resource that systematically analyses the infiltration of various immune cells and their clinical impact across a spectrum of cancer types [20]. In this study, it was used for the analyses of *PTEN* expression in correlation with immune infiltrating cells, including CD8 + and CD4 + T cells, by the purity-corrected partial Spearman method. p-values < 0.05 were considered statistically significant.

### Statistical analysis

Statistical analyses were performed using SPSS for Windows version 26.0 (SPSS, Chicago, IL, USA). Normality was evaluated using Kolmogorov-Smirnov and Shapiro-Wilk tests. Non-parametric tests Mann-Whitney, Kruskal-Wallis, and ANOVA were used to compare distributions of variables. A p-value of < 0.05 (two-sided) was considered as statistically significant.

## Results

### Study group

From February 2019 to July 2022, a total of 36 patients diagnosed with grade IV GBM were recruited for this study. Tissue samples were obtained from 23 primary and 13 recurrent tumours. Altogether, the study group consisted of 13 female and 23 male GBM patients; more patient demographics and baseline characteristics are given in Table 1. Baseline characteristics of patients between the wild-type and mutant PTEN genotypes were similar.

**Table 1:** Patient demographics and baseline characteristics

Characteristic		Total	PTEN WT	PTEN Mutant
Age, years, no (%)	≤ 50	8 (22.2%)	8 (50.0%)	0
	> 50	28 (77.8%)	8 (50.0%)	20 (100%)
Gender, no (%)	Female	13 (36.1%)	6 (37.5%)	7 (35.0%)
	Male	23 (63.9%)	10 (62.5%)	13 (65.0%)
Karnofsky Performance Scale (KPS)	100	11 (30.6%)	3 (18.75%)	8 (40.0%)
	90	9 (25.0%)	4 (25.0%)	5 (25.0%)
	80	8 (22.2%)	4 (25.0%)	4 (20.0%)
	70	3 (8.3%)	2 (12.5%)	1 (5.0%)
	≤ 60	5 (13.9%)	3 (18.75%)	2 (10.0%)
Site of target lesion(s)	Temporal	12 (33.3%)	3 (18.75%)	9 (45.0%)
	Frontal	11 (30.5%)	6 (37.5%)	5 (25.0%)
	Parietal	6 (16.7%)	4 (25.0%)	2 (10.0%)
	Occipital	4 (11.1%)	2 (12.5%)	2 (10.0%)
	Temporo-occipital	2 (5.6%)	1 (6.25%)	1 (5.0%)
	Other	1 (2.8%)	0	1 (5.0%)
Resection	Gross total	32 (88.9%)	14 (87.5%)	18 (90%)
	Subtotal	3 (8.3%)	2 (12.5%)	1 (5.0%)
	Biopsy*	1 (2.8%)	0	1 (5.0%)
Invasion	No	14 (38.9%)	6 (37.5%)	8 (40.0%)
	Corpus callosum	11 (30.5%)	5 (31.25%)	6 (30.0%)
	Multiple	1 (2.8%)	0	1 (5.0%)
	Multifocal	4 (11.1%)	3 (18.75%)	1 (5.0%)
	Multifocal + corpus callosum	3 (8.3%)	1 (6.25%)	2 (10.0%)
	Brainstem + corpus callosum	1 (2.8%)	0	1 (5.0%)
	Other	2 (5.6%)	1 (6.25%)	1 (5.0%)

*3 cc incisional (via mini-craniotomy) biopsy sample

### PTEN mutations and expression profile

Coding regions of the *PTEN* gene were sequenced in a total of 36 tissue samples. No mutations were detected in 15 (41.67%) tumour samples; whereas, a total of 28x *PTEN* mutations were identified in the other 21 (58.33%) tumour samples. As shown in Supplementary Table 1; 15x missense, 6x frameshift, 5x start/stop loss, 1x stop gained, and 1x synonymous mutation were found and classified according to the VarSome database.

**Fig. 1:**
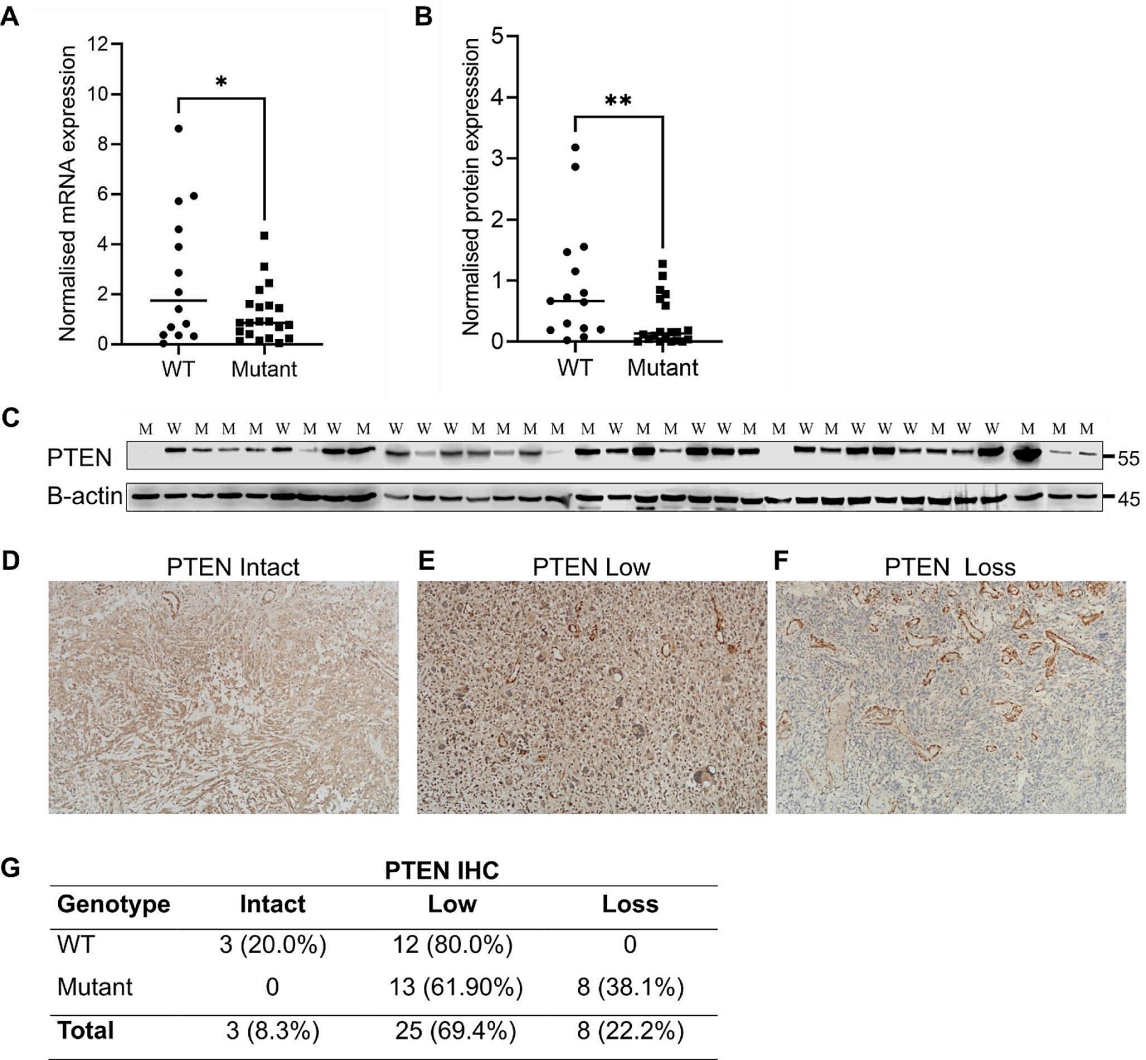
PTEN expression status in wild-type (WT) and mutant (M) genotypes. **(A)** qRT-PCR results of *PTEN* mRNA expression normalised to *GAPDH* (*p = 0.008); **(B)** Western Blot analysis of PTEN protein expression normalised to B-actin (**p = 0.006); **(C)** Western Blot images of PTEN in the GBM tissues. *PTEN* genotypes are indicated as WT and M at the top of the gel; **(D)** Immunohistochemistry analysis of PTEN showing intact expression; **(E)** Weaker staining of PTEN with focal loss. Cases were clustered as PTEN low; **(F)** PTEN immunohistochemistry image demonstrates complete PTEN loss, images at 100x magnification; **(G)** Comparison of PTEN IHC expression in relation to *PTEN* genotypes

Mutant *PTEN* patients had significantly lower PTEN expression, both at mRNA (p = 0.008) and protein (p = 0.006) levels (Fig. 1c-e). For one sample, the tissue lysate did not work for western blot analyses; therefore, protein expression was only evaluated by IHC. IHC analysis showed that whereas PTEN expression in 3 patients (5.6%) was intact; decreased expression could be detected in 26 patients (72.2%) and complete loss in 8 patients (22.2%) (Fig. 1f-g). All patients with PTEN loss carried the mutant *PTEN* genotype. Besides, PTEN expression did not significantly change between primary and recurrent tumours (Supp. File, Fig. 1).

### Effects of PTEN status on the cGAS-STING pathway

**Fig. 2:**
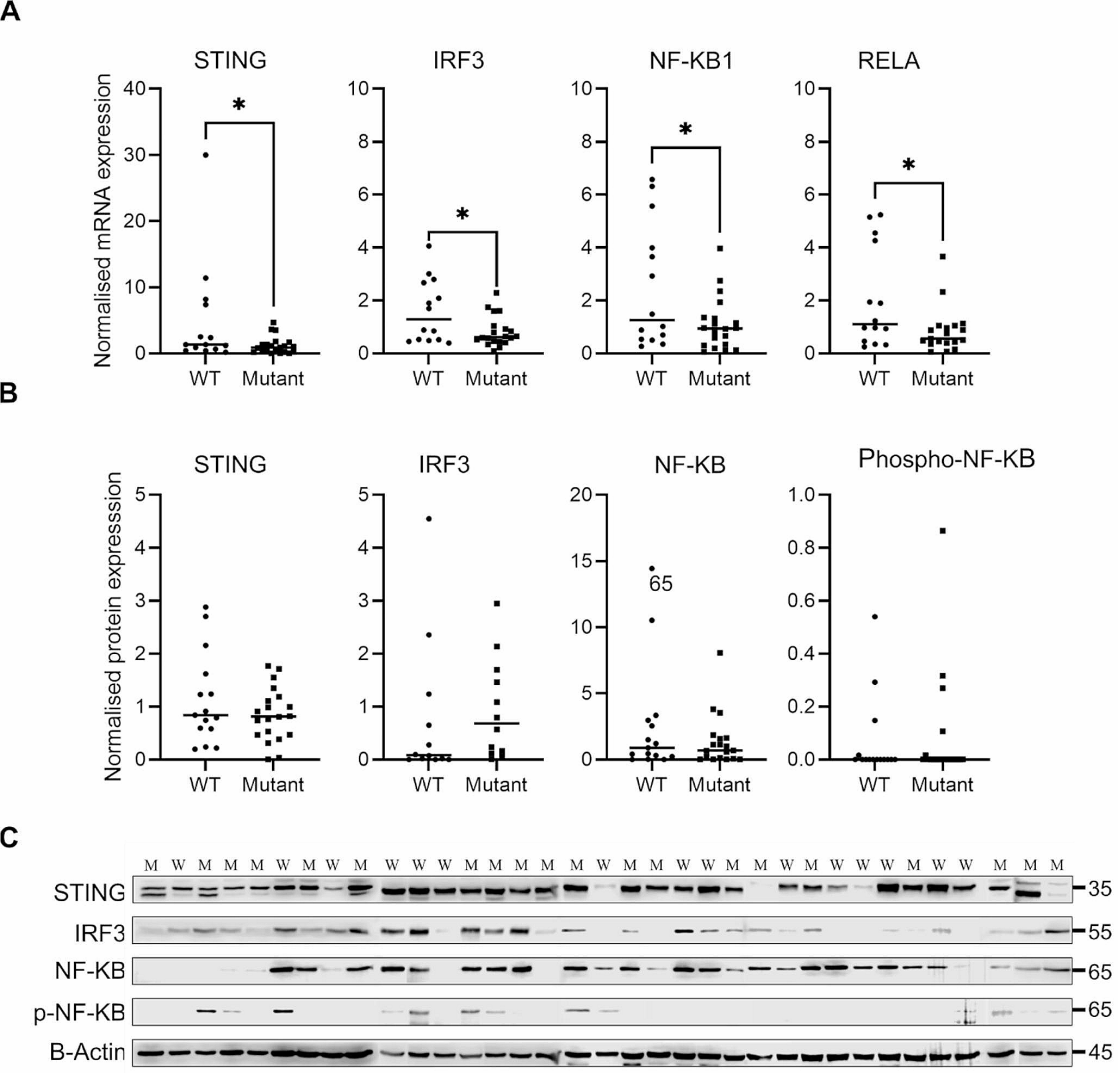
Effects of *PTEN* genotypes on cGAS-STING pathway. **(A)** mRNA expression graphs of *STING, IRF3, NF-KB1*, and *RELA* genes in wild-type (WT) and mutant (M) patients; **(B)** Protein expressions of STING, IRF3, NF-KB, and Phospho-NF-KB normalized to B-actin; **(C)** Western Blot images of protein expressions. *PTEN* genotypes are indicated as WT and M at the top of the gel

The expression patterns of cGAS-STING pathway members in GBM tissue samples were compared in relation to primary and recurrent tumours, and to *PTEN* genotypes of the patients. Accordingly, mRNA expressions did not significantly differ between primary and recurrent tumour samples (Supp. File, Fig. 1). Interestingly, *STING, IRF3, NF-KB1*, and *RELA* mRNA expressions decreased significantly in patients with the mutant *PTEN* genotype (Fig. 2a). However, protein expressions did not significantly differ between patients with wild-type or mutant genotypes (Fig. 2b-c).

### Effects of PTEN expression onto tumour immunogenicity

**Fig. 3:**
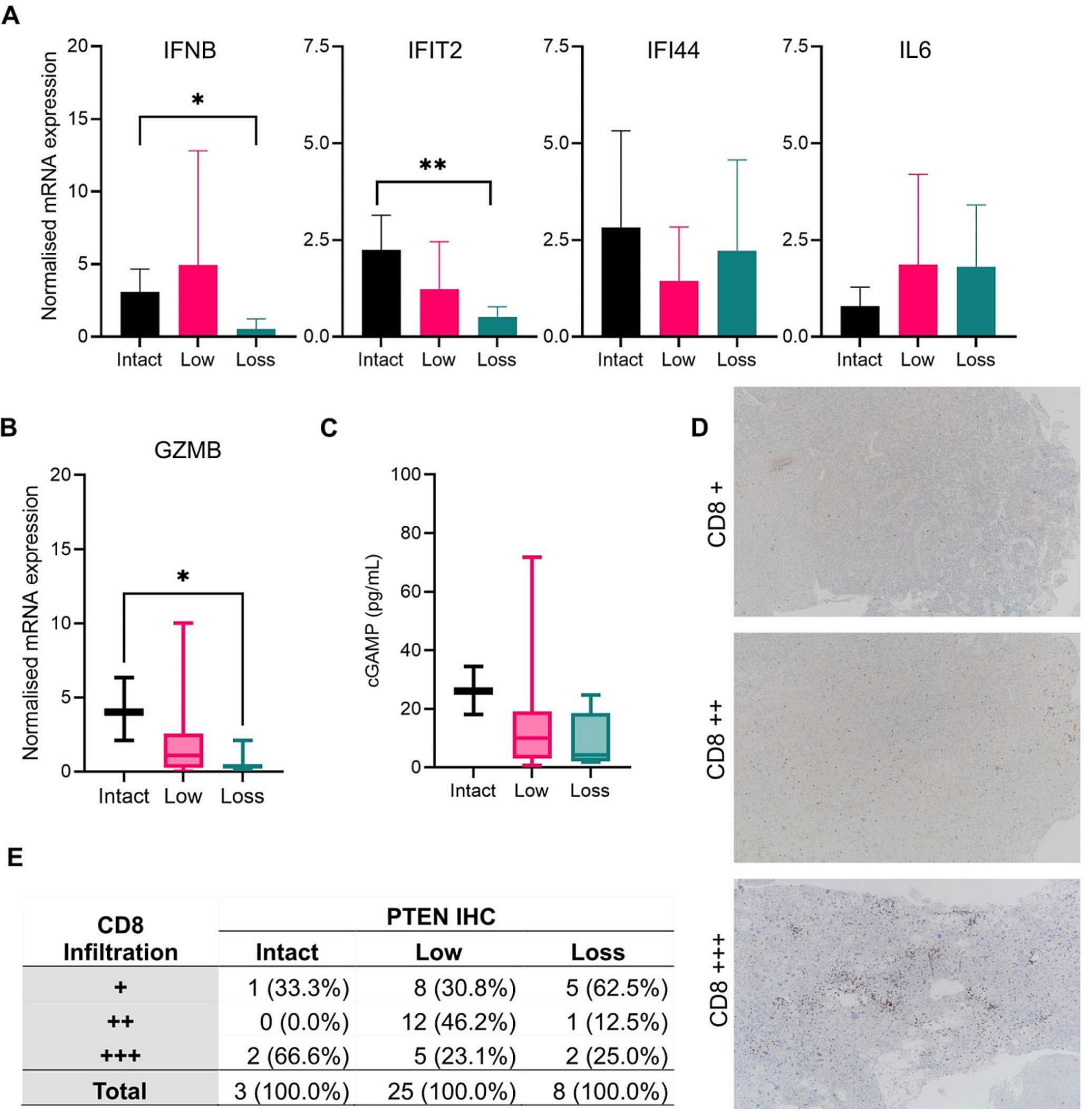
Effects of PTEN expression to tumour immunogenicity. **(A)** Transcription levels of interferon-stimulated genes according to immunohistochemically expression status of PTEN (*: p = 0.033; **: p = 0.015); **(B)** Normalised mRNA expression of *GZMB* (*: p = 0.045); **(C)** cGAMP production measured by ELISA in tumour lysates; **(D)** Immunohistochemistry analysis of CD8 showing 3 grades of infiltration. **E**: Comparison of CD8 infiltration in relation to PTEN status, images at 40x magnification

For evaluating the role of PTEN expression on cGAS-STING activity and tumour immunogenicity, mRNA expression levels of interferon-stimulated genes, especially *IFNB, IFIT2, IFI44*, and *IL6* were analysed. The results showed that *IFNB* (p = 0.033) and *IFIT2* (p = 0.015) mRNA levels significantly decreased in patients with PTEN loss (Fig. 3a). Consistently, Granzyme B (*GZMB*) expression levels also decreased in patients with PTEN loss (p = 0.045) (Fig. 3b). Analyses of cGAMP production in whole tumour protein lysates revealed a wide range of cGAMP expression levels in GBM tumour samples (Fig. 3c). Although not statistically significant, mean cGAMP was lowest in patients with PTEN loss.

To reveal if CD8 infiltration is altered in relation with PTEN expression, tumour samples were immunohistochemically stained with anti-CD8 antibody and evaluated by two pathologists. Considering the staining intensity, three groups could be established (Fig. 3d). Samples of patients with PTEN loss showed significantly decreased CD8 infiltration (p = 0.04) (Fig. 3e).

**Fig. 4:**
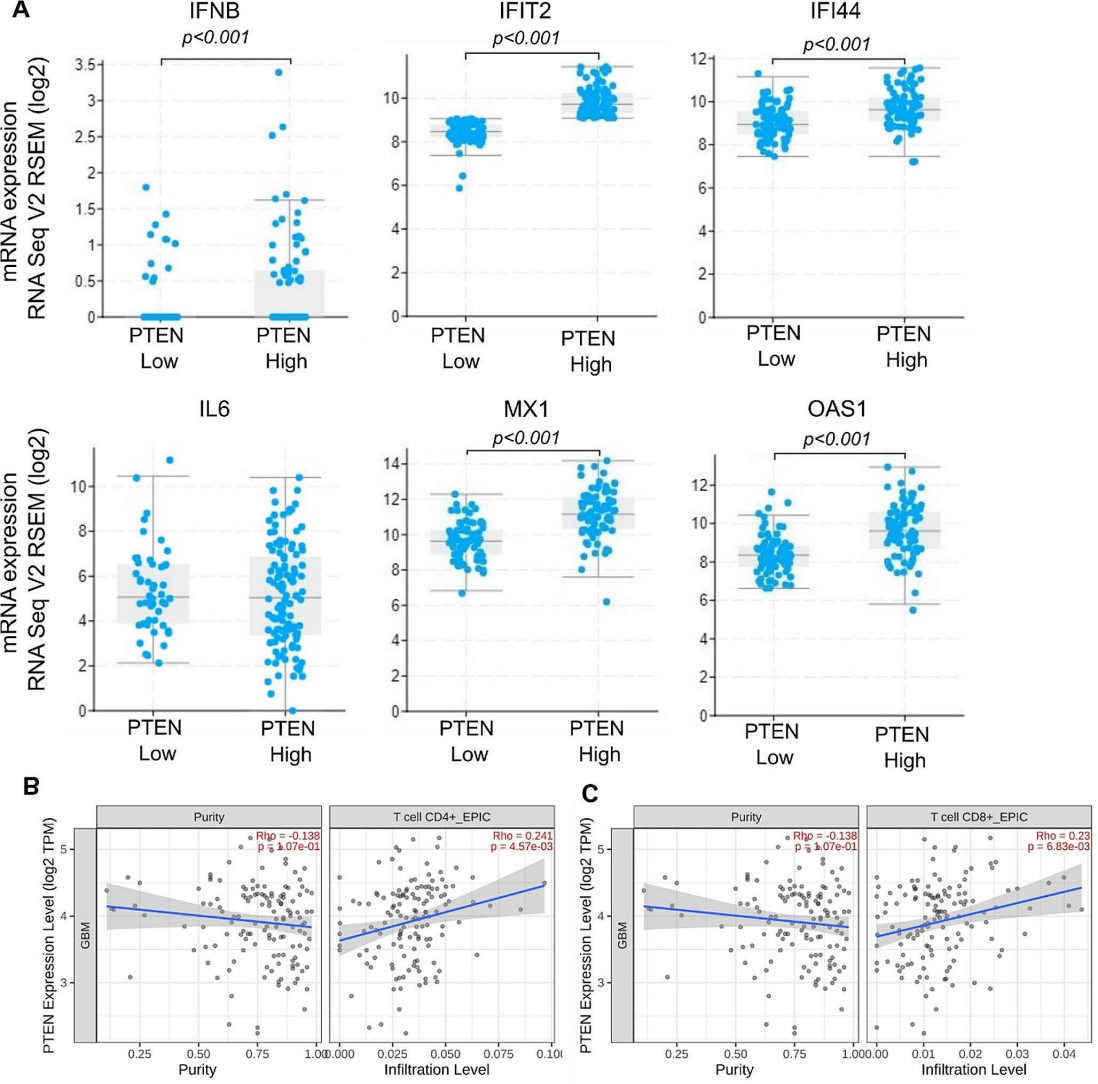
TCGA database analysis. Median PTEN expression was used to discriminate low (n = 81) and high (n = 81) expression groups. **(A)** mRNA expression levels of interferon-stimulated genes; **(B)** CD4+; and **(C)** CD8 + cell infiltration in relation to PTEN expression

Bioinformatics analysis was performed on data from 162 IDH negative GBM samples obtained from the TCGA database for validation studies. Different ISG genes, such as *MX1* and *OAS*, and infiltration of additional immune cells were also investigated and similar results obtained; i.e., the low *PTEN* mRNA expressing group displayed significantly decreased ISGs (*IFNB*, *IFIT2*, *IFI44*, *MX1*, and *OAS1*); and, CD4 + and CD8 + cell infiltration (Fig. 4a-c).

## Discussion

Immunosuppressive effects of PTEN have been studied extensively in several tumours. However, the mechanism of these effects has not been fully established to date. This study reports 28x *PTEN* mutations in 21 (58.33%) GBM tumour samples and its significantly low mRNA and protein expression levels in those *PTEN* mutant tumours. A GBM prognostic model based on immune-regulated genes revealed that patients with *PTEN* mutation are in the high-risk group [21]. Increased immune cell infiltration, more aggressive immune activity, higher expression of immune checkpoint genes, and less benefit from immunotherapy are listed among the characteristic features of these high-risk group patients [21]. These results are also in accordance with the PTEN-associated immune prognostic signature in GBM [22, 23].

Does *PTEN* mutations effect tumour immunogenicity through the cGAS-STING pathway? It is known that PTEN has an essential phosphatase activity on IRF3, a master transcription factor responsible for IFNB synthesis [13]. *PTEN* mutations in the catalytic phosphatase domain can impair phosphatase activity, either by gain or loss of function; some mutations can even effect just lipid, but not protein phosphatase activity [24, 25]. Therefore, mutation-dependent gain or loss of function of phosphatase activity might have potential effects on interferon release. This study shows that expression of cGAS-STING pathway members like *STING, IRF3, NF-KB1*, and *RELA* indeed decreases in patients carrying the mutant *PTEN* genotype.

In a recently published article, the presence of STING has particularly been shown in the tumour vasculature in human GBM samples [26]. Several reports also highlighted the positive immunogenic effects of STING agonist treatments in the GBM microenvironment [26–30]. Circulation of the STING protein through the vesicular traffic pathway includes sorting into Rab7-positive endolysosomes before degradation [31]. Rab7 and TBK1, on the other side, are substrates of PTEN, by which way it can contribute to the vesicular traffic of STING [32]. Disruption of STING trafficking, regulated by the Rab7 and TBK1 axis, in breast cancer cells with PTEN loss also supports the role of PTEN on tumour immunogenicity through the cGAS-STING pathway [32].

PTEN deficiency induces galectin-9 secretion, which drives M2 macrophage polarization and therefore is associated with angiogenesis and glioma progression [33]. A recent study showed that macrophage PTEN deficiency regulates nuclear factor (erythroid-derived 2)-like 2 (NRF2) function, and interrelates with NICD before it controls STING-mediated TBK1 function, resulting in enhanced inflammatory response in liver injury [34]. Both studies indicate that PTEN loss may also influence STING regulation in the tumour microenvironment by affecting macrophage polarization. However, further studies are needed to elucidate the mechanism of how tumour-specific regulations occur.

Contributions of PTEN to tumour immunity through the cytosolic cGAS-STING pathway may provide new insights into the treatment of PTEN loss tumours. For instance, PTEN-null triple-negative breast cancer (TNBC) cell lines were hyper-responsive to STING agonists because of diminished STING turnover and increased production of IRF3 targets [32]. Recently, it has been shown that PTEN deficiency in high-grade serous ovarian cancer resulted in decreased sensitivity to carboplatin alone. However, STING activation potentiates response to carboplatin chemotherapy, prolongs overall survival, repolarizes M2-like suppressive macrophages, and rescues T-cell activation in the tumour microenvironment [35]. Due to the immune privilege of the brain, GBM has a “cold tumour” phenotype, and displays low numbers of tumour-infiltrating lymphocytes and other immune effector cell types. This current study shows that PTEN-deficient GBM patients have decreased CD8 infiltration, Granzyme B activity, and interferon expression. Bioinformatics analysis using the TCGA database also revealed decreased ISGs and T cell infiltration in GBM patients, validating our findings. Therefore, STING activation might turn the GBM microenvironment into a “hot tumour” to benefit from improved immunotherapy.

Nevertheless, there are some limitations of this study. Surgical operations and follow-ups of patients have not solely been accomplished by Ege University Faculty of Medicine. Because of this, data was not accessible for all patients and survival analyses could not be performed. However, the main aim of this study was to determine immunogenic effects that might occur by PTEN loss through the cGAS-STING pathway, rather than its survival effects. Another limitation is the low number of cases included into this study, as we hope that soon other studies will support the relationship between PTEN and cGAS-STING in GBM [36].

In conclusion, immunosuppressive effects of PTEN loss through the cGAS-STING pathway in GBM tumours could be shown in this study. The future perspective of this study would be if these results could be applied to translational studies for the clinical treatment of PTEN-null tumours. Finally, we propose that the PTEN protein is required for the proper functioning of the cGAS-STING pathway and that STING agonists may not be effective in GBM tumours with PTEN loss.

### Electronic supplementary material

Below is the link to the electronic supplementary material.

Supplementary Material 1

## Data Availability

No datasets were generated or analysed during the current study.
