# Supplementary material for: Investigating the effects of PTEN mutations on cGAS-STING pathway in glioblastoma tumours
Source: J Neurooncol. 2024 Jan 12;166(2):283–92. doi: 10.1007/s11060-023-04556-4 (PMC10834568; doi:10.1007/s11060-023-04556-4)
Supplement: Supplementary file 1 — Supplementary Material 1 [file 11060_2023_4556_MOESM1_ESM.docx]

Supplementary Material for

**Investigating the effects of PTEN mutations on cGAS-STING pathway**

**in glioblastoma tumours**

**Supplementary Table 1:** *PTEN* mutations detected in GBM tumour samples

| Sample No | Chr 10  position | Verdict | Coding impact | Variant type | HGVC | AA | IHC |
| --- | --- | --- | --- | --- | --- | --- | --- |
| 1 | 87864400 | Pathogenic | Frameshift | Del | (NM_001304717.5):c.452_455del | p.Lys151SerfsTer23 | Loss |
| 2 | 87864471 | Pathogenic | Start loss | SNV | (ENST00000371953.8):c.2T>G | p.Met1Arg | Loss |
| 2 | 87894051 | Pathogenic | Missense | SNV | (NM_001304717.5):c.625G>A | p.Gly209Arg | Loss |
| 2 | 87933084 | Pathogenic | Missense | SNV | (NM_001304717.5):c.844G>A | p.Asp282Asn | Loss |
| 3 | 87960991 | Pathogenic | Frameshift | Del | (NM_001304717.5):c.1419de | p.Ile473MetfsTer7 | Loss |
| 4 | 87952142 | Pathogenic | Missense | Del | (NM_001304717.5):c.1036C>T | p.Arg346Cys | Low |
| 5 | 87864400 | Pathogenic | Frameshift | Del | (NM_001304717.5):c.452_455del | p.Lys151SerfsTer23 | Loss |
| 5 | 87925550 | Pathogenic | Missense | SNV | (NM_001304717.5):c.721T>C | p.Tyr241His | Loss |
| 6 | 87960919 | Pathogenic | Missense | SNV | (NM_001304717.5):c.1346A>G | p.Asn449Ser | Low |
| 7 | 87957958 | Pathogenic | Frameshift | Ins | (NM_001304717.5):c.1260dup | p.Pro421ThrfsTer5 | Loss |
| 8 | 87864522 | Pathogenic | Missense | SNV | (NM_001304717.5):c.572A>G | p.Glu191Gly | Low |
| 8 | 87933073 | Pathogenic | Missense | SNV | (NM_001304717.5):c.833G>T | p.Cys278Phe | Low |
| 9 | 87864400 | Pathogenic | Frameshift | Del | (NM_001304717.5):c.452_455del | p.Lys151SerfsTer23 | Loss |
| 9 | 87952173 | US-LP | Missense | SNV | (NM_001304717.5):c.1067A>G | p.Lys356Arg | Loss |
| 10 | 87864471 | Pathogenic | Start loss | SNV | (ENST00000371953.8):c.2T>G | p.Met1Arg | Low |
| 11 | 87864514 | Pathogenic | Missense | SNV | (NM_001304717.5):c.564A>T | p.Arg188Ser | Low |
| 12 | 87894091 | Pathogenic | Missense | SNV | (NM_001304717.5):c.665A>T | p.Asn222Ile | Low |
| 13 | 87957915 | Pathogenic | Stop Gained | SNV | (NM_001304717.5):c.1216C>T | p.Arg406Ter | Low |
| 14 | 87957974 | Pathogenic | Frameshift | Ins | (NM_001304717.5):c.1276dup | p.Ile426AsnfsTer45 | Loss |
| 15 | 87864461 | Benign | Missense | SNV | (NM_001304717.5):c.511C>G | p.Leu171Val | Low |
| 16 | 87864343 | US-B | Synonymous | SNV | (NM_001304717.5):c.393G>A | p.Ala131= | Low |
| 16 | 87864471 | Pathogenic | Start loss | SNV | (ENST00000371953.8):c.2T>G | p.Met1Arg | Low |
| 17 | 87864514 | Pathogenic | Missense | SNV | (NM_001304717.5):c.564A>T | p.Arg188Ser | Low |
| 18 | 87965471 | Pathogenic | Stop loss | SNV | (NM_001304717.5):c.1730G>T | p.Ter577Leuext*8 | Low |
| 19 | 87933061 | Pathogenic | Missense | SNV | (NM_001304717.5):c.821T>C | p.Ile274Thr | Loss |
| 20 | 87864471 | Pathogenic | Start loss | SNV | (ENST00000371953.8):c.2T>G | p.Met1Arg | Low |
| 21 | 87933156 | Pathogenic | Missense | SNV | (NM_001304717.5):c.916G>A | p.Val306Ile | Low |
| 21 | 87933166 | Pathogenic | Missense | SNV | (NM_001304717.5):c.926G>A | p.Cys309Tyr | Low |

*HGVC: Human Genome Variation Society, AA: Amino Acid, IHC:* *[Immunohistochemistry of PTEN](https://en.wikipedia.org/wiki/Immunohistochemistry), SNV: Single Nucleotide Variant, Del: Deletion, Ins: Insertion, US-LP: Uncertain Significance-Likely Pathogenic, US-B: Uncertain Significance-Benign*


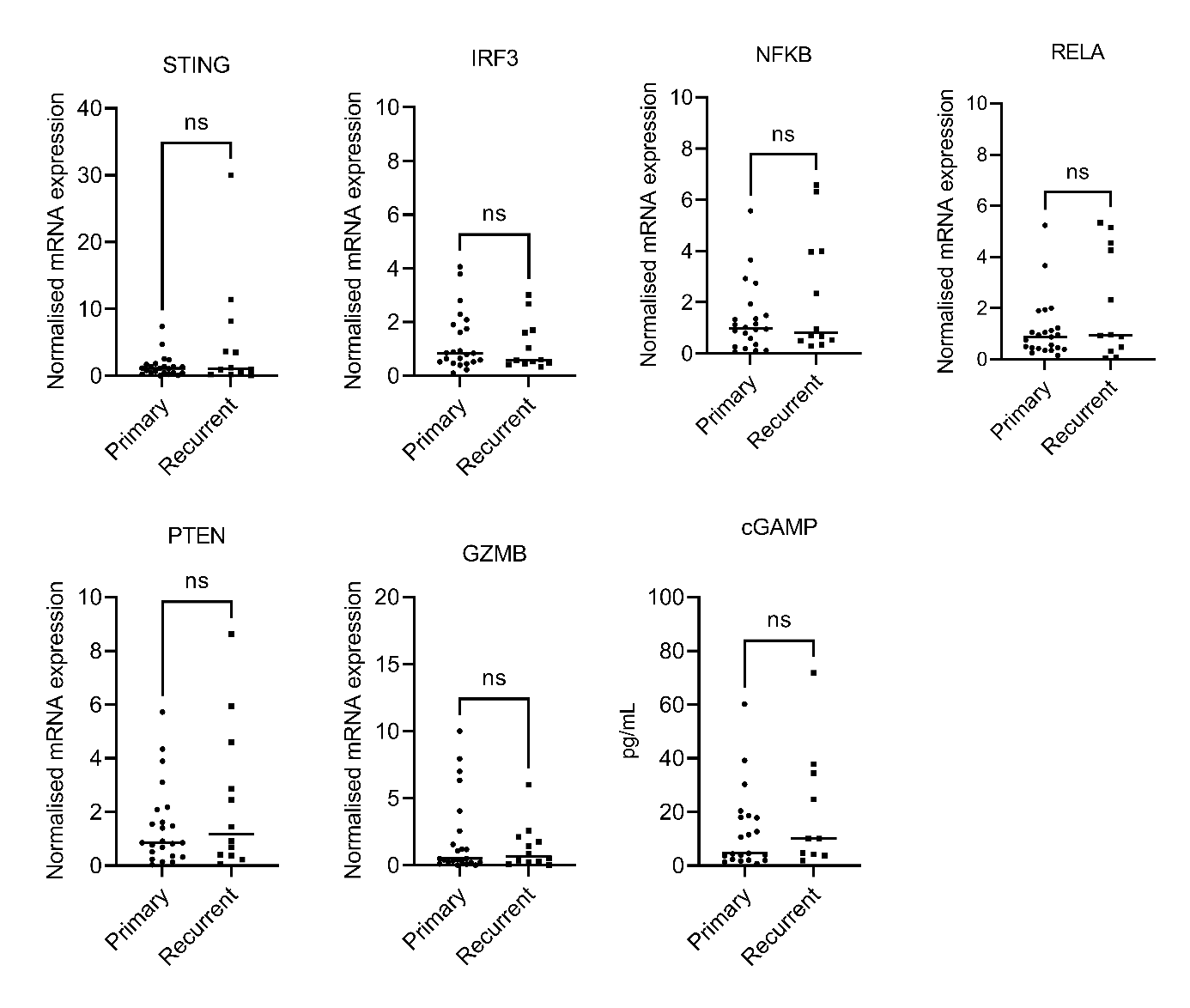


**Supplementary Figure 1.** Comparison of gene expression profiles of primary and recurrent tumours; ns: not significant.


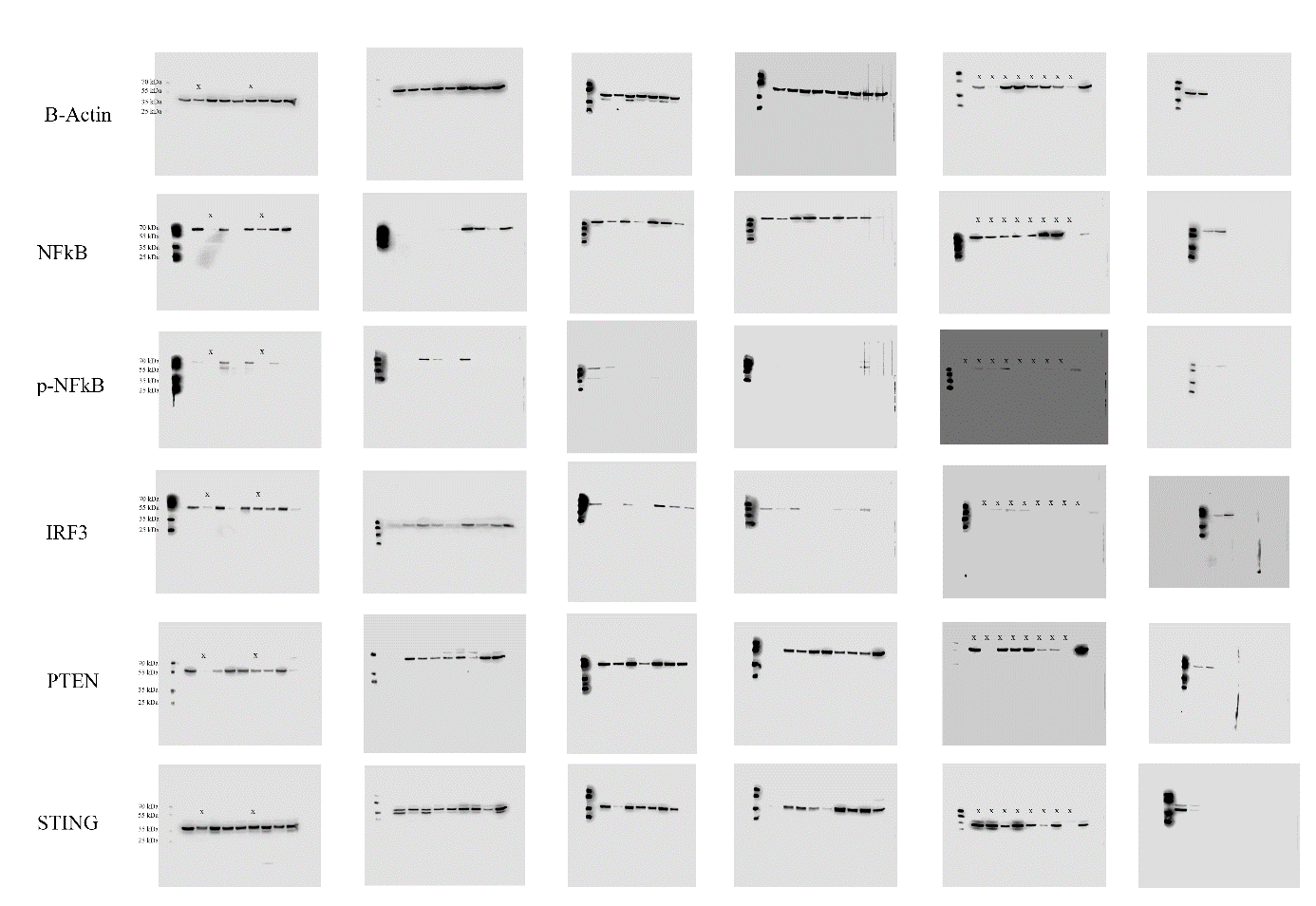


**Supplementary Figure 2:** Original western blot images of target proteins. The first line in all gels shows the pre-stained protein ladder (Fermentas, #SM0671). The WesternSure Pen is used to detect protein ladder prior to chemiluminescent detection (Li-Cor). “X” indicates patients that were excluded from this study, either because of their samples different histological type or insufficient concentration.
